# Longevity extension of worker honey bees (*Apis mellifera*) by royal jelly: optimal dose and active ingredient

**DOI:** 10.7717/peerj.3118

**Published:** 2017-03-28

**Authors:** Wenchao Yang, Yuanyuan Tian, Mingfeng Han, Xiaoqing Miao

**Affiliations:** 1 Apitherapy Institute, College of Bee Science, Fujian Agriculture and Forestry University, Fuzhou, China; 2 Bee Product Processing and Application Research Center of the Ministry of Education, Fuzhou, China

**Keywords:** Worker bee, Bee pollen, Royal jelly, RJP_60_, Longevity

## Abstract

In the Western honey bee, *Apis mellifera*, queens and workers have different longevity although they share the same genome. Queens consume royal jelly (RJ) as the main food throughout their life, including as adults, but workers only eat worker jelly when they are larvae less than 3 days old. In order to explore the effect of RJ and the components affecting longevity of worker honey bees, we first determined the optimal dose for prolonging longevity of workers as 4% RJ in 50% sucrose solution, and developed a method of obtaining long lived workers. We then compared the effects of longevity extension by RJ 4% with bee-collected pollen from rapeseed (*Brassica napus*). Lastly, we determined that a water soluble RJ protein obtained by precipitation with 60% ammonium sulfate (RJP_60_) contained the main component for longevity extension after comparing the effects of RJ crude protein extract (RJCP), RJP_30_ (obtained by precipitation with 30% ammonium sulfate), and RJ ethanol extract (RJEE). Understanding what regulates worker longevity has potential to help increase colony productivity and improve crop pollination efficiency.

## Introduction

Royal jelly (RJ) is a yellow or pale syrup-like substance which is secreted by the hypopharyngeal and mandibular glands of young honey bees (“nurses”) mainly for feeding the queen larvae and adults (Fujita et al., 2012). Worker destined larvae (younger than 3 days old) will develop into queen if they are fed with RJ in queen cells. The composition of RJ varies slightly with season and geographical origin (Zheng, Hu & Dietemann, 2010). Chemically, RJ is composed of 60–70.0% water, 9–18% protein, 3–8% lipids, 3–13% fructose, 4–8% glucose, 0.5–2.0 % sucrose, 0.8–3% ash, and 1.4–2.01% 10-hydroxy-2-decenoic acid (10-HDA) (Tamura et al., 2009; Sabatini et al., 2009; Wei et al., 2013). The nine most abundant proteins, which by weight account for 80% of soluble proteins, have been termed as major RJ proteins (MRJPs) (Simuth, 2001).

Caged honey bees have been used to study many factors affecting longevity (reviewed by Williams et al., 2013), for example, by *Nosema* infection (Milbrath et al., 2015) and nutrition (Schmidt, Thoenes & Levin, 1987; Wu, Anelli & Sheppard, 2011; Paoli et al., 2014; Li et al., 2014). The main nutrition sources for honey bees are nectar or honey and pollen, which provides protein, lipids and vitamins (Yang et al., 2013). Longevity extension of caged workers is observed when bees are provided with bee-collected pollen (Schmidt, Thoenes & Levin, 1987; Wang et al., 2014). The suitable amount of proteins or amino acid consumed in their diet is positively correlated with longevity (Standifer, 1967; De Groot, 1953). Workers fed with pollen and RJ survive better than those fed with pollen only (Wang et al., 2014).

Queen and worker honey bees both develop from fertilized eggs; their phenotypical differences are solely due to differences in larval rearing environment, especially nutrition. Workers and queens are fed jelly with similar composition during in the first three days of larval development but significantly different later (Haydak, 1943). Queens eat RJ throughout their whole life, while adult workers feed on honey and bee pollen. The average life expectancy of queen bees is more than 20 times of that of worker bees (Page Jr & Peng, 2001). As a result of nutritional differences during larval stages, vitellogenin levels, juvenile hormone titers and insulin signaling are different in queens and workers, creating the difference in longevity (Corona et al., 2007). Queen larvae have lower DNA methylation levels compared to worker larvae (Foret et al., 2012; Lyko et al., 2010), perhaps as a main mechanism to cause different phenotypes (Wang et al., 2014; Shi et al., 2011; Kucharski et al., 2008). Previous studies determined that RJ has longevity extension effects on C3H/HeJ mice (Inoue et al. 2003), fruit flies (Chen et al., 2009; Chang, Guo & Ma, 2008; Kamakura, 2011; Xiao, 2013), and nematode *C. elegans* (Honda et al., 2011).

RJ and its components can affect activity and longevity of worker bees. Worker brain levels of tyrosine, dopamine and tyramine and ovarian development were improved when they were fed with RJ (Lin & Winston, 1998; Matsuyama, Nagao & Sasaki, 2015). Feeding workers with tyrosine, a component of RJ, inhibited behavioral development (more in-hive proportion than sucrose only group) and promoted ovarian development (Matsuyama, Nagao & Sasaki, 2015). Low dose of RJ extends longevity of caged workers, but worker mortality is increased at high doses (Pirk et al., 2010; Paoli et al., 2014). Caged bees die within 3 days when they are fed with 100% RJ (no honey) (Paoli et al., 2014), and within 5 days when fed with a diet of casein, RJ, and sucrose, with a ratio of protein to carbohydrate at 3:1 (Pirk et al., 2010). Royalactin, major royal jelly protein 1 (MRJP1), induces differentiation of larvae to be queen (Kamakura, 2011), but there is also a controversy (Buttstedt et al., 2016; Kamakura, 2016). A substance similar to RJ is distributed to all adult workers and drones (Crailsheim, 1991).

The optimal protein to carbohydrate ratio (P: C) is determined as 1:14 using RJ and sucrose over a 14 day experiment (Altaye et al., 2010). However it is not clear what is the optimal RJ dose for long term survival in honey bee workers, though whole RJ does have longevity extension effects on female fruit flies (Chang, Guo & Ma, 2008) and nematode *C. elegans* (Honda et al., 2011). Substances prolonging longevity of male or female fruit flies are MRJPs (Xiao, 2013), which can be separated by precipitation with 30% and 60% saturated ammonium sulfate solution (Salazar-Olivo & Paz-Gonzalez, 2005). RJ hydrolyzed by Protease N and 10-HDA are also longevity-promoting factors for nematode *C. elegans* (Honda et al., 2011). We hypothesize that (a) there is an optimized dose of RJ for workers; and (b) MRJPs and 10-HDA (included in ethanol extract of RJ) will prolong worker longevity. This study attempts to determine the optimal dose of RJ and its main component(s) (ingredients of MRJPs separated by ammonium sulfate solution and ethanol extract of RJ) on longevity extension for caged honey bees. The results would lay the foundation for further development and utilization of RJ. Effective ingredient(s) can be fed in an apiary to increase both honey production and pollination efficiency in summer or increased number of workers over winter.

## Materials and Methods

### Chemical determination and composition separation of RJ

#### Chemical determination of RJ

The main chemical components of RJ (provided by the Fujian Provincial Shenfeng Science and Technology Development Co., Ltd., harvested in Zhejiang region with canola flower in Spring 2013), such as protein, moisture, acidity, ash, total sugar and 10-HDA were measured by HPLC method according to GB9697-2008 (GAQSIQ, 2008). The experiments below were carried out in Fuzhou, Fujian, China, May 2013 to Jan 2014.

### Fractioning of RJ proteins

A solution of RJ (20%, 200 g RJ added to a total of 1,000 ml 0.05 M pH = 7.5 phosphate buffered saline (PBS, 1L aqueous solution containing 250 ml 0.2 M PBS, 0.5585 g NaCl, and 7.4400 g ethylene diamine tetra-acetic acid)), was prepared and centrifuged at 12,000 g, 4 °C for 30 min. The supernatant was dialyzed with 0.2 M Tris–HCl (pH 7.5) for 24 h and lyophilized. We termed this lyophilize powder as RJ crude proteins (RJCP) (Srisuparbh et al., 2003). RJCP also was used for further separation using ammonium sulfate.

RJP_30_ and RJP_60_ was separated by precipitation of 30% and 60% saturated ammonium sulfate solution from RJCP. Briefly, RJP_30_ was precipitated first by 30% ammonium sulfate, then the supernatant was used for RJP_60_ which was precipitated with 60% saturated ammonium sulfate. These are different from the methods by Salazar-Olivo & Paz-Gonzalez (2005), who precipitated RJ proteins with 30% (RJP_30_) or 60% (RJP_60_) ammonium sulfate directly. In other words, our two fractions were serial and theirs were parallel, so their RJP_60_ contains fractions of RJP_30_, while ours does not. Saturated ammonium sulfate solution (188 ml, 3.8 M) was added into 400 ml RJCP solution (thus creating a solution of ammonium sulfate that is 30% of saturation), and stored under 4 °C for 1 h. The solution was centrifuged at 12,000 g, 4 °C for 30 min. The pellet was dissolved and dialyzed with reverse osmosis (RO) water (14 kDa dialysis membrane, Beijing Branch Hongda Biotechnology Co. Division) for 24 h, and the content inside the bag was dried with a lyophilizer. This fraction is termed “RJP_30_”. The supernatant (588 ml) was precipitated with 3.8 M saturated ammonium sulfate solution (493 ml), and then stored at 4 °C for 1 h. The solution was centrifuged at 12,000 g, 4 °C for 30 min. The pellet was dissolved and dialyzed with RO water (14 kDa dialysis bag) for 24 h and the content inside the bag was dried by a lyophilizer. The powder obtained was termed “RJP_60_”, because the ammonium sulfate concentration was 60% of a fully saturated solution.

RJP_30_ and RJP_60_ were compared by 8% sodium dodecyl sulfate—Polyacrylamide gel electrophoresis (SDS-PAGE) (Hames, 1998). Briefly, 8 % gradient gel with a 3.5% stacker was prepared in the Bio-Rad Mini Protean III Cell using 1-mm spacers. Samples of RJP_30_ and RJP_60_ (each with 7.5 μg protein, determined by Bio-Rad method) and marker (5 µl, Prestained Protein Ladder, Beijing Solarbio Science & Technology Co., Ltd.) were loaded. Electrophoresis begun with voltage set at 60 V and increased to 100 V when the dye front had run into the gradient gel. After the dye front had run off the gel, the gel was picked up and stained by staining solution (0.1 g Coomassie Brilliant Blue G-250, 10 g ammonium sulfate, 2 mL 85% phosphoric acid and water added to 500 mL) for 10 min at room temperature. Then the gel was distained in a distaining solution (37.5 mL glacial acetic acid, 25 mL of methanol and water added to 500 mL) for about 3 h until the protein was clear.

### RJ ethanol extract

RJ was dissolved in 6 volumes of 95% ethanol and shaken for 60 min in a shaking water bath (45 °C). The mixture was centrifuged at 5,000 g for 10 min. The pellet was concentrated by a rotary evaporator and then dried in a vacuum to obtain RJ ethanol extract (RJEE), which included 10-HDA, other fatty acids and ethanol soluble proteins.

The yields of RJP_30_, RJP_60_, RJCP and RJEE were weighted using analytical balance (CP214, Ohaus Instrument (Shanghai) Co. Ltd; China).

### Optimal dose of RJ on longevity extension

In order to determine the optimal dose of RJ on worker longevity, this experiment was designed. Sealed brood frames were selected from three healthy colonies (raised in the apiary of Apitherapy Institute, Fujian Agriculture and Forestry University, Fuzhou, China, GPS coordinates: 119.232 and 26.085) and placed in a constant temperature and humidity chamber (35 °C, RH 75%). After 24 h, 1-day-old workers were obtained and put in cages (50 bees per cage) maintained at 35 °C and RH 50%. Cages (90  × 80 × 110 mm) were made with wood and a slideable plexiglass door with holes drilled. There were two feeding holes on the top of each cage, which were used to fix two 15ml glass feeding bottles, one for RO water and one for 50% sucrose solution or 50% sucrose solution with additive, such as RJ if necessary. Both water and 50% sucrose solution or 50% sucrose solution with additive were replaced every two days. In order to explore the optimal RJ dose for longevity-extending effects, five treatments (sucrose, bovine serum albumin (BSA), 2%, 4% and RJ 16%) with 3 caged bees (from 3 different colonies, 1 cage bee from 1 colony) per treatment were used. BSA was used as a protein control because RJ contained mainly proteins. Dead workers were removed from each cage daily till the 39th day, when the trials were terminated.

### Effects of RJ and pollen on longevity extension

To determine whether the effects of longevity extension by RJ were due to a general effect of extra protein or a specific effect of RJ, we compared the effects of longevity extension by the RJ dose determined in the last experiment and that of bee-collected pollen, a normal protein source of workers, from rapeseed (*Brassica campestris* L.). This species pollen was identified by using the same colored pollen pellets which were microscopically matched to the pollen collected directly from the plant. Bee pollen, sucrose and water (2:1:1, weight) were mixed and soaked for 24 h, and then was kept about 9 g mixture in a plastic cap 10 mm × 30 mm (height × inner diameter). The plastic cap was hung with a wire in the cage. The pollen diets of workers were fed *ad libitum* and changed daily. RO water and 50% sucrose solution in two feeding bottles were provided via two feeding holes and were changed daily. Four treatments were used in this experiment, which were Sucrose: 5.0 g 50% sucrose solution; BSA: 0.12 g BSA + 4.88 g 50% sucrose solution; *Brassica: Brassica* bee pollen + 50% sucrose solution and RJ 4%: 0.2 g RJ + 4.8 g 50% sucrose solution. Each test was repeated three times using bees from three different colonies. Dead workers were removed daily till the 72nd day.

### Longevity extension by RJ components

In order to observe the longevity extension effects of RJ components, six treatments were designed according to the yield of four fractions and the optimal RJ dose, which were RJP_30_: 0.017 g RJP_30_ + 4.983 g 50% sucrose solution; RJEE: 0.034 g RJEE + 0.75 g ethanol + 4.216 g 50% sucrose solution; Sucrose: 0.75 g ethanol + 4.25 g 50% sucrose solution; RJCP: RJ crude protein 0.289 g + 4.711 g 50% sucrose solution; RJP_60_: 0.023 g RJP_60_ + 4.977 g 50% of sucrose solution and RJ 4%: 0.2 g RJ + 4.8 g 50% sucrose solution. Each treatment had three cages with bees from three different colonies. Dead workers were removed every day till the 72nd day.

### Statistical analyses

The results were described with means and standard errors. Survival analyses (Log-Rank) of caged honey bees (*Apis mellifera*) was used to determine differences in longevity among different treatments by using R version 3.3.1 for windows (The R Foundation for Statistical Computing).

## Results

### Chemical composition and isolated RJ components

Water content of RJ used throughout this study was determined to be 63.5 ± 0.06%, protein 14.2 ± 0.11%, total sugar 14.5 ± 0.18%, ash 1.4 ± 0.05%, acidity 37 ± 0.21 mL/100 g and 10-HDA 1.7 ± 0.04% (*n* = 3 determinations). These physical and chemical features of RJ were within the ranges specified by the Chinese National Standard (GAQSIQ, 2008).

**Figure 1:**
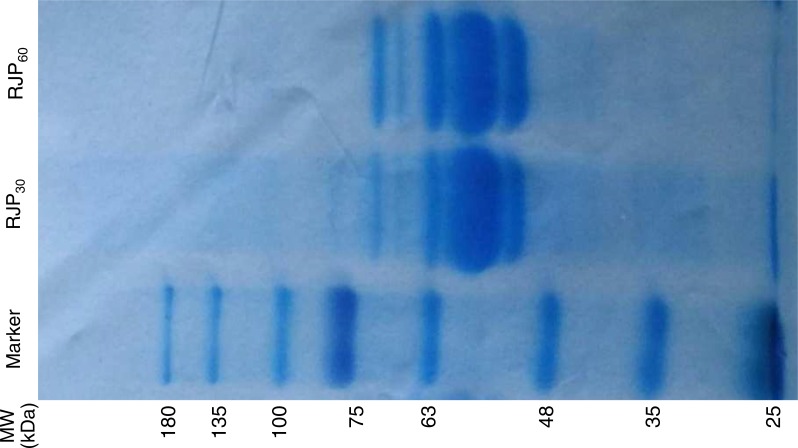
SDS-PAGE electrophoresis of RJP_30_ and RJP_60_ (7.5 µg). MW: molecular weight.

The yields (dry weight of each fraction divided by dry weight of protein in RJ in the starting material) of RJCP, RJP_30_, RJP_60_ and RJEE were 17.43 ± 0.07%, 1.50 ± 0.03%, 17.06 ± 0.09% and 15.40 ± 0.07% respectively. The proteins separated by electrophoresis in RJP_30_ and RJP_60_ are shown in Fig. 1. Both fractions have similar profiles, but RJP_60_ has more 70 kDa protein than RJP_30_, which has more proteins with >70 kDa.

### The optimal RJ dose on longevity extension

**Figure 2:**
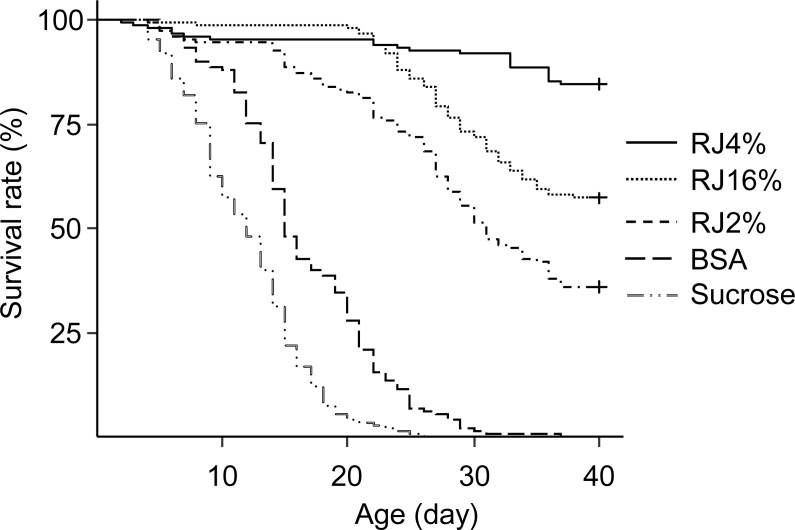
Survival curves of caged honey bees (*Apis mellifera*) fed with different doses of royal jelly. Each cage contained 50 bees; each treatment had 3 cages from 3 colonies.

The differences in worker longevity among treatments were significant (Fig. 2, Survival analysis, Log-Rank Test, *X*^2^ = 836, *df* = 4, *P* < 0.0001). There were significant differences in longevity between workers in all pairwise comparisons between the adjacent survival curves (*X*^2^ = 16.4–171, *P* ≪ 0.001). Bees fed RJ 4% had the longest lifespan, followed by bees fed RJ 16%, RJ 2% and BSA, with those fed sucrose dying the fastest.

### Longevity extension by RJ and rapeseed pollen

**Figure 3:**
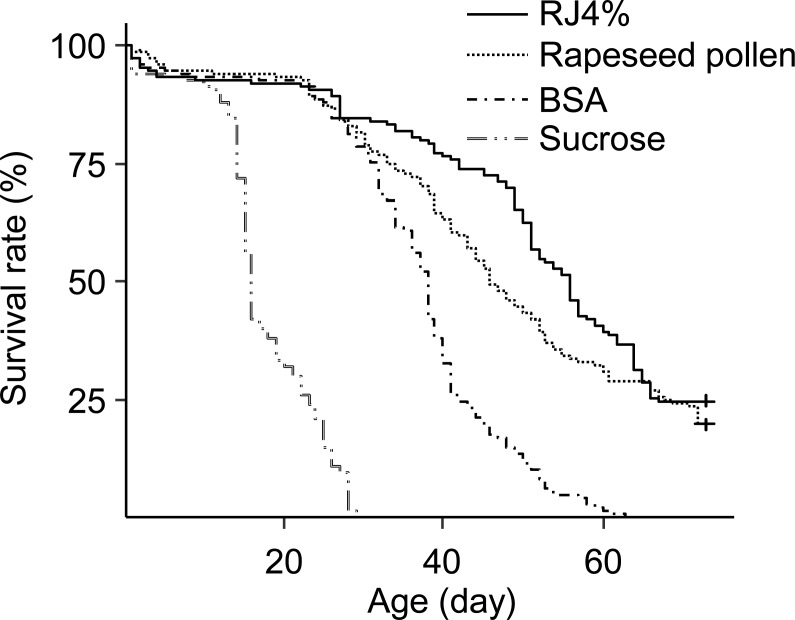
Survival curves of caged honey bees (*Apis mellifera*) fed with rapeseed (*Brassica*) bee-collected pollen and 4% RJ. Each cage contained 50 bees; each treatment had 3 cages from 3 colonies.

The differences in worker longevity among treatments were significant (Fig. 3, Survival analysis, Log-Rank Test, *X*^2^ = 610, *df* = 3, *P* < 0.0001). The longest survival was found in bees fed RJ 4%, followed by bees fed *Brassica* pollen, BSA, and sucrose only. All pairwise comparisons between the adjacent survivals curves were significant (*X*^2^ = 4.4–240, *P* < 0.05).

### Longevity extension by RJ components

**Figure 4:**
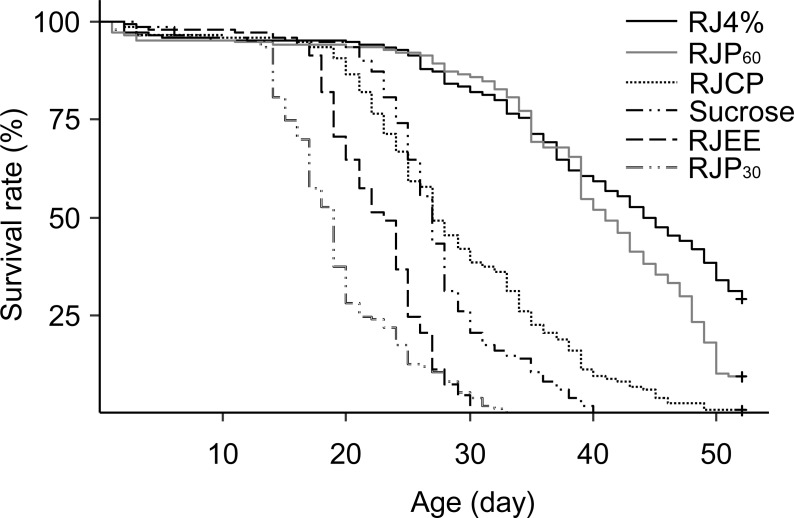
Survival curves of caged honey bees (*Apis mellifera*) fed with different royal jelly fractions. Each cage contained 50 bees; each treatment had 3 cages from 3 colonies.

The differences in worker longevity among treatments were highly significant (Fig. 4, Log Rank Test, *X*^2^ = 752, *df* = 5, *P* < 0.0001). The best survival was found in bees fed RJ 4%, followed by RJP_60_, RJCP, 50% sucrose, RJEE, and RJP_30_ in descending order. All pairwise comparisons between the adjacent survivals curves were significant (*X*^2^ = 10.9–106, *P* < 0.001).

## Discussion

The main finding of this study is that 4% royal jelly in sugar syrup has the best effect on extending the longevity of workers (Fig. 2). That RJ can prolong the lifespan of workers has been well-documented (Altaye et al., 2010; Pirk et al., 2010; Paoli et al., 2014; Wang et al., 2014). Lifespan of workers fed with 50% pollen and 50% RJ were longer than those workers fed with pollen only (Wang et al., 2014). But the quantity of RJ eaten by workers is unclear. Altaye et al. (2010) found that there was no difference between longevity of workers fed with RJ 2% in 50% sucrose solution and RJ 4% in 50% sucrose solution over a 14 day experiment. The results of our experiment (39 days) showed that the best longevity extension of RJ in 50% sucrose solution is 4%. If we analyze our data only at 14 days there was also no difference in survival between RJ 2% and 4% (*X*^2^ = 1.94, *df* = 1, *P* = 0.16), consistent with Altaye et al. (2010). At this concentration (4%), carbohydrate meets the energetic needs of honey bees, and RJ provides protein for workers. But higher doses of RJ reduced longevity in our study, similar to Paoli et al. (2014) which found bees fed with high ratio of essential amino acids to carbohydrate (≥1:10) diets had shorter lifespans than those fed diets containing low levels of dietary amino acids. The reason of high mortality might be due to too much protein in the diet, which can cause toxic effects due to nitrogen metabolism.

We also found that the main effect for longevity extension of RJ is due to proteins inside RJP_60_. The longevity extending effect of RJ is not solely due to the extra protein it provides because BSA treatment provided similar protein amount but caused a much smaller effect in longevity extension. BSA increased mean worker longevity by 4.66 days compared to sucrose but RJ 4% more than doubled worker lifespan (Figs. 2 and 3). Because BSA treatment also lacked lipids and vitamins, it is not clear whether RJ caused the longest survival due to protein plus lipids and vitamins. We therefore compared RJ with bee collected *Brassica* pollen in extending worker longevity. The longevity extension effect of RJ 4% was better than this bee collected pollen (Fig. 3). RJP_60_ showed almost the same longevity extension effect as that of RJ 4% (Fig. 4). We conclude that the RJP_60_ fraction contains the active protein ingredient(s) for longevity extension. Previous studies discovered that RJ or its fractions could prolong the longevity of animals. The average lifespan of mice fed with RJ at 6 mg/kg and 60 mg/kg had their lifespan extended by about 25% (Chen et al., 2009). The fifth fraction of octadecyl-silica column chromatography of protease-treated RJ extended the longevity of *C. elegans* (Pirk et al., 2010). The pantothenic acid in RJ prolonged the lifespan of *Drosophila melanogaster* (Gardner, 1948). As far as we know, this study is the first one to determine both the optimal dosage of RJ in 50% sucrose solution and show a crude separation of active ingredients in extending longevity in honey bee workers.

RJP_30_ and RJP_60_ were separated with a different method from a prior study (Salazar-Olivo & Paz-Gonzalez, 2005). In that study, RJP_30_ and RJP _60_ were precipitated with 30% (RJP_30_) and 60% (RJP_60_) ammonium sulfate from RJCP directly (parallel), so that RJP_60_ will contain the same proteins of RJP_30_. In this study, RJP_30_ was precipitated first by 30% ammonium sulfate, then the supernatant was used for RJP_60_ which was precipitated with 60% saturated ammonium sulfate. Even though there were equal *molecular weight* (MW) proteins in them, especially 48–63 and 70 kDa MW proteins, there were proteins >75 or <48 kDa in RJP30 but not in RJP_60_. In addition, the yield of RJP_30_ was only about 8.8% of RJP_60_. These differences may have caused the different longevity-extending effects.

Worker longevity is influenced by pollen nutrition. Mixture of pollens have a higher nutritive value and can extend longevity of caged bees more compared to providing pollen from a single species (Haydak, 1970). For example, a five-pollen mix (an equal mix of *Cereus, Larrea, Populus, Prosopias* and *Prunus* bee pollen) increased LT50 (50% of longevity, the age at which 50% of bees still remained alive) by 40.6 days while the best single pollen *Populus* increased LT50 by 38 days, with all other single pollen causing only <20 days of LT50 increase, over the baseline of 20 days (sugar only) (Schmidt, Thoenes & Levin, 1987). Other times, mixed pollen do not increase LT50 because a single species of pollen is already highly nutritious. *Rubus* pollen is highly nutritious and an equal mix of pollen from *Rubus, Castanea, Erica, and Cistus* did not differ in longevity from *Rubus* pollen alone (Di Pasquale et al., 2013). In our study, *Brassica* pollen, the best nutritional bee pollen which can prolong longevity 2.5 times relative to the controls (sucrose only) compared with Sunflower pollen 1.6 times and Sesame pollen 1.7 times (Schmidt et al., 1995), was employed to compare the longevity prolonging effects of RJ 4% (Fig. 3). The lipid and protein contents in *Brassica* are 6.65% and 27–29% separately (Gardner, 1948). The protein content in the sucrose solution of RJ 4% group was only 0.57%, much lower than that of pollen, yet it extended worker lifespan more. This suggests that RJ protein maybe of higher quality and may not even need processing (digestion). The LT50 of our bees fed with *Brassica* bee pollen were 45 (Fig. 4) to 55 days (Fig. 3), this is comparable to the longest LT50 of bees provided with 5 pollen mix (60 days Schmidt et al., 1995) and that of rapeseed pollen (50 days Schmidt et al., 1995). The LT50 from Fig. 2 was not estimable because survival was over 80% when that experiment was terminated at 40 days.

Bee pollen also affects worker health and gland development. Adequate nutrition is important for the development of healthy honey bee colonies (Brodschneider & Crailsheim, 2010). Both individual and social immunocompetence changed with different pollen diets, but increased dietary protein did not enhance immunocompetence (Alaux et al., 2010). Bees fed with polyfloral pollen diets had higher glucose oxidase activity than those fed with monofloral diets (Alaux et al., 2010). Different pollen, sugar or commodity diets affected the development of both venom glands and hypopharyngeal glands (Omar et al., 2016). size of hypopharyngeal glands are shown to have a strong positive correlation with oocyte length (Wegener et al., 2009) in caged honey bees. High protein bee pollen enhances ovarian development of caged bees (Hoover, Higo & Winston, 2006; Human et al., 2007), but for *Apis mellifera capensis* it inhibits ovarian development (Schäfer et al., 2006).

The three experiments (Figs. 2–4), with bees from 9 different colonies, showed different longevity for the sucrose control, ranging from 11 to 27 days. This could be due to differences in time of year, colony conditions or due to colony genetics.

Inside colonies, honey bee workers can live much longer when they are changed to *diutinus* (long-lived bees, also called winter bees) bees. Maurizio (1950) created long lived bees during summer by caging the queen to create broodless conditions. These long lived bees are found to be caused mainly by a lack of brood pheromone, with bees exposed to neither brood nor brood pheromone living to over 200 days. Workers with either brood or brood pheramones have intermediate longevity and those with both have the shortest longevity (Smedal et al., 2009). It is possible that conditions inside cages never truly replicate that inside a colony, so that workers can only live to 120 days inside cages (extrapolated from LT50 of 60 days to maximum longevity).

RJP_30_ showed a toxicity to worker bees, with a shorter longevity of bees than the control (sucrose only) (Fig. 4). This fraction (although prepared slightly differently, see above) was also shown to be cytotoxic for HeLa human cervicouterine carcinoma cells (Salazar-Olivo & Paz-Gonzalez, 2005). In the test of longevity-extending effects of RJ components (Fig. 4), 0.75 g ethanol was added to RJEE and sucrose only treatments (4.25 g), but no ethanol was added in other groups. The LT50 of RJEE is lower than sucrose solution, but both of which were higher than that of RJP_30_. These results suggest that the lower LT50 of RJEE was not caused by ethanol in diet.

Differences in worker longevity can greatly affect colony productivity. Longer lived adult workers enabled a larger population, a longer foraging life duration (Becerra-Guzmán et al., 2005) and increased honey yield (Botías et al., 2012) and hence also higher pollination capacity (Harbo, 1986). Understanding how to prolong worker life span can therefore be useful for increasing both honey production and pollination efficiency if any food supplement can be used inside colonies in a field setting. RJP_60_ with sucrose solution can be fed to colonies to prolong longevity of workers and then increase both honey production and pollination efficiency in summer or increase proportion of long-lived workers during winter. It is possible that there is a benefit (increased honey production) to the colony due to increased survival but there might be also a cost (e.g., extra energy required to produce the RJ), thus explaining the natural “norm” we now observe. However, if we can artificially manipulate this set point at the colony level, there might be a net gain.

##  Supplemental Information

10.7717/peerj.3118/supp-1 Supplemental Information 1 Database of survival Raw data

10.7717/peerj.3118/supp-2 Supplemental Information 2 R code for survival analysis

10.7717/peerj.3118/supp-3 Supplemental Information 3 Supplemental Table 1 and Figures 1–3

## Additional Information and Declarations

### Data Availability

The following information was supplied regarding data availability: The raw data has been supplied as a Supplementary File.
