# Supplementary material for: Longevity extension of worker honey bees (Apis mellifera) by royal jelly: optimal dose and active ingredient
Source: PeerJ. 2017 Mar 28;5:e3118. doi: 10.7717/peerj.3118 (PMC5372980; doi:10.7717/peerj.3118)
Supplement: Supplemental Information 2 [file peerj-05-3118-s002.docx]

library(survival)

install.packages("ggplot2", dependencies=TRUE)

ggsurv <- function(s, CI = 'def', plot.cens = T, surv.col = 'gg.def',

cens.col = 'red', lty.est = 1, lty.ci = 2,

cens.shape = 3, back.white = F, xlab = 'Time',

ylab = 'Survival', main = ''){

library(ggplot2)

strata <- ifelse(is.null(s$strata) ==T, 1, length(s$strata))

stopifnot(length(surv.col) == 1 | length(surv.col) == strata)

stopifnot(length(lty.est) == 1 | length(lty.est) == strata)

ggsurv.s <- function(s, CI = 'def', plot.cens = T, surv.col = 'gg.def',

cens.col = 'red', lty.est = 1, lty.ci = 2,

cens.shape = 3, back.white = F, xlab = 'Time',

ylab = 'Survival', main = ''){

dat <- data.frame(time = c(0, s$time),

surv = c(1, s$surv),

up = c(1, s$upper),

low = c(1, s$lower),

cens = c(0, s$n.censor))

dat.cens <- subset(dat, cens != 0)

col <- ifelse(surv.col == 'gg.def', 'black', surv.col)

pl <- ggplot(dat, aes(x = time, y = surv)) +

xlab(xlab) + ylab(ylab) + ggtitle(main) +

geom_step(col = col, lty = lty.est)

pl <- if(CI == T | CI == 'def') {

pl + geom_step(aes(y = up), color = col, lty = lty.ci) +

geom_step(aes(y = low), color = col, lty = lty.ci)

} else (pl)

pl <- if(plot.cens == T & length(dat.cens) > 0){

pl + geom_point(data = dat.cens, aes(y = surv), shape = cens.shape,

col = cens.col)

} else if (plot.cens == T & length(dat.cens) == 0){

stop ('There are no censored observations')

} else(pl)

pl <- if(back.white == T) {pl + theme_bw()

} else (pl)

pl

}

ggsurv.m <- function(s, CI = 'def', plot.cens = T, surv.col = 'gg.def',

cens.col = 'red', lty.est = 1, lty.ci = 2,

cens.shape = 3, back.white = F, xlab = 'Time',

ylab = 'Survival', main = '') {

n <- s$strata

groups <- factor(unlist(strsplit(names

(s$strata), '='))[seq(2, 2*strata, by = 2)])

gr.name <- unlist(strsplit(names(s$strata), '='))[1]

gr.df <- vector('list', strata)

ind <- vector('list', strata)

n.ind <- c(0,n); n.ind <- cumsum(n.ind)

for(i in 1:strata) ind[[i]] <- (n.ind[i]+1):n.ind[i+1]

for(i in 1:strata){

gr.df[[i]] <- data.frame(

time = c(0, s$time[ ind[[i]] ]),

surv = c(1, s$surv[ ind[[i]] ]),

up = c(1, s$upper[ ind[[i]] ]),

low = c(1, s$lower[ ind[[i]] ]),

cens = c(0, s$n.censor[ ind[[i]] ]),

group = rep(groups[i], n[i] + 1))

}

dat <- do.call(rbind, gr.df)

dat.cens <- subset(dat, cens != 0)

pl <- ggplot(dat, aes(x = time, y = surv, group = group)) +

xlab(xlab) + ylab(ylab) + ggtitle(main) +

geom_step(aes(col = group, lty = group))

col <- if(length(surv.col == 1)){

scale_colour_manual(name = gr.name, values = rep(surv.col, strata))

} else{

scale_colour_manual(name = gr.name, values = surv.col)

}

pl <- if(surv.col[1] != 'gg.def'){

pl + col

} else {pl + scale_colour_discrete(name = gr.name)}

line <- if(length(lty.est) == 1){

scale_linetype_manual(name = gr.name, values = rep(lty.est, strata))

} else {scale_linetype_manual(name = gr.name, values = lty.est)}

pl <- pl + line

pl <- if(CI == T) {

if(length(surv.col) > 1 && length(lty.est) > 1){

stop('Either surv.col or lty.est should be of length 1 in order

to plot 95% CI with multiple strata')

}else if((length(surv.col) > 1 | surv.col == 'gg.def')[1]){

pl + geom_step(aes(y = up, color = group), lty = lty.ci) +

geom_step(aes(y = low, color = group), lty = lty.ci)

} else{pl + geom_step(aes(y = up, lty = group), col = surv.col) +

geom_step(aes(y = low,lty = group), col = surv.col)}

} else {pl}

pl <- if(plot.cens == T & length(dat.cens) > 0){

pl + geom_point(data = dat.cens, aes(y = surv), shape = cens.shape,

col = cens.col)

} else if (plot.cens == T & length(dat.cens) == 0){

stop ('There are no censored observations')

} else(pl)

pl <- if(back.white == T) {pl + theme_bw()

} else (pl)

pl

}

pl <- if(strata == 1) {ggsurv.s(s, CI , plot.cens, surv.col ,

cens.col, lty.est, lty.ci,

cens.shape, back.white, xlab,

ylab, main)

} else {ggsurv.m(s, CI, plot.cens, surv.col ,

cens.col, lty.est, lty.ci,

cens.shape, back.white, xlab,

ylab, main)}

pl

}

test3 <- read.csv("filename.csv",head=TRUE, sep=",")

#test3=subset(test3, trt!="A") #if necessary,delete "#"

#test3=subset(test3, trt!="B")#if necessary,delete "#"

#test3=subset(test3, trt!="C")#if necessary,delete "#"

#test3=subset(test3, trt!="D")#if necessary,delete "#"

#test3=subset(test3, trt!="E")#if necessary,delete "#"

#test3=subset(test3, cage==1) #keeping only some data, here only for colony 1. #if necessary,delete "#"

test2=NULL

newline=NULL

for (i in 1:nrow(test3))

{

for (j in 1:test3[i,3]) test2=rbind(test2, test3[i,c(1,2,4,5)])

}

bee.surv2 <- survfit(Surv(age,censor) ~ trt, data = test2)

(pl2 <- ggsurv(bee.surv2))

survdiff(Surv(age,censor)~trt, data=test2, rho=0)
