# Supplementary material for: Longevity extension of worker honey bees (Apis mellifera) by royal jelly: optimal dose and active ingredient
Source: PeerJ. 2017 Mar 28;5:e3118. doi: 10.7717/peerj.3118 (PMC5372980; doi:10.7717/peerj.3118)
Supplement: Supplemental Information 3 [file peerj-05-3118-s003.doc]

**Supplemental Table 1:**

ANOVA among colonies

| Groups | **Colony** | **X^2^** | **p** |
| --- | --- | --- | --- |
| RJ | **Total** | **20.1** | **<0.0001** |
|  | **1 VS 2** | **20.1** | **<0.0001** |
|  | **1 VS 3** | **2.2** | **0.138** |
|  | **2 VS 3** | **8.9** | **0.0029** |
| Pollen | **Total** | **10.9** | **0.004** |
|  | **1 VS 2** | **3.3** | **0.071** |
|  | **1 VS 3** | **2** | **0.158** |
|  | **2 VS 3** | **11.6** | **0.0007** |
| Components of RJ | **Total** | **9** | **0.011** |
|  | **1 VS 2** | **2.6** | **0.105** |
|  | **1 VS 3** | **9.6** | **0.0019** |
|  | **2 VS 3** | **1.5** | **0.223** |

**Supplemental Fig 1A:**

The differences in longevity among the 3 colonies were significant between colony 1 and colony 2 (X^2^ = 20.1, df = 1, *P* < 0.0001), and colony 2 and colony 3 (X^2^ = 8.9, df = 1, *P* = 0.003). Colony 1 did not resolve the differences among the first three treatments (RJ 4% = RJ 16% = RJ 2%) but the following treatments were in the same order (RJ2% > BSA) (Fig A in S1 Figs). Colony 2 and 3 were consistent in having the order of worker longevity as RJ 4% > RJ 16% > RJ 2% > BSA > sucrose (Fig B and C in S1 Figs).


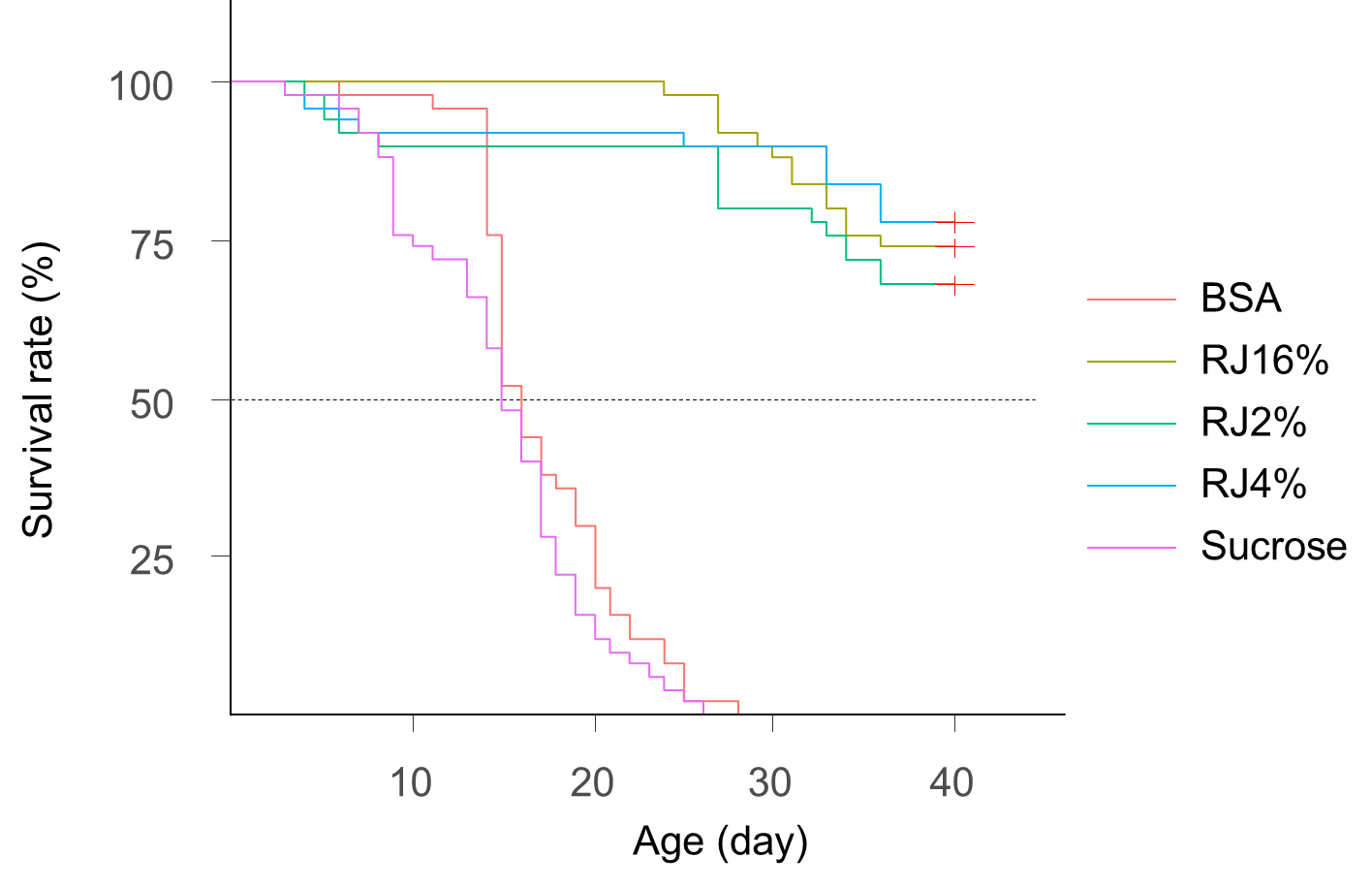


Colony 1: (RJ4% = RJ 16% = RJ2% > BSA = sucrose)

**Supplemental Fig 1B:**


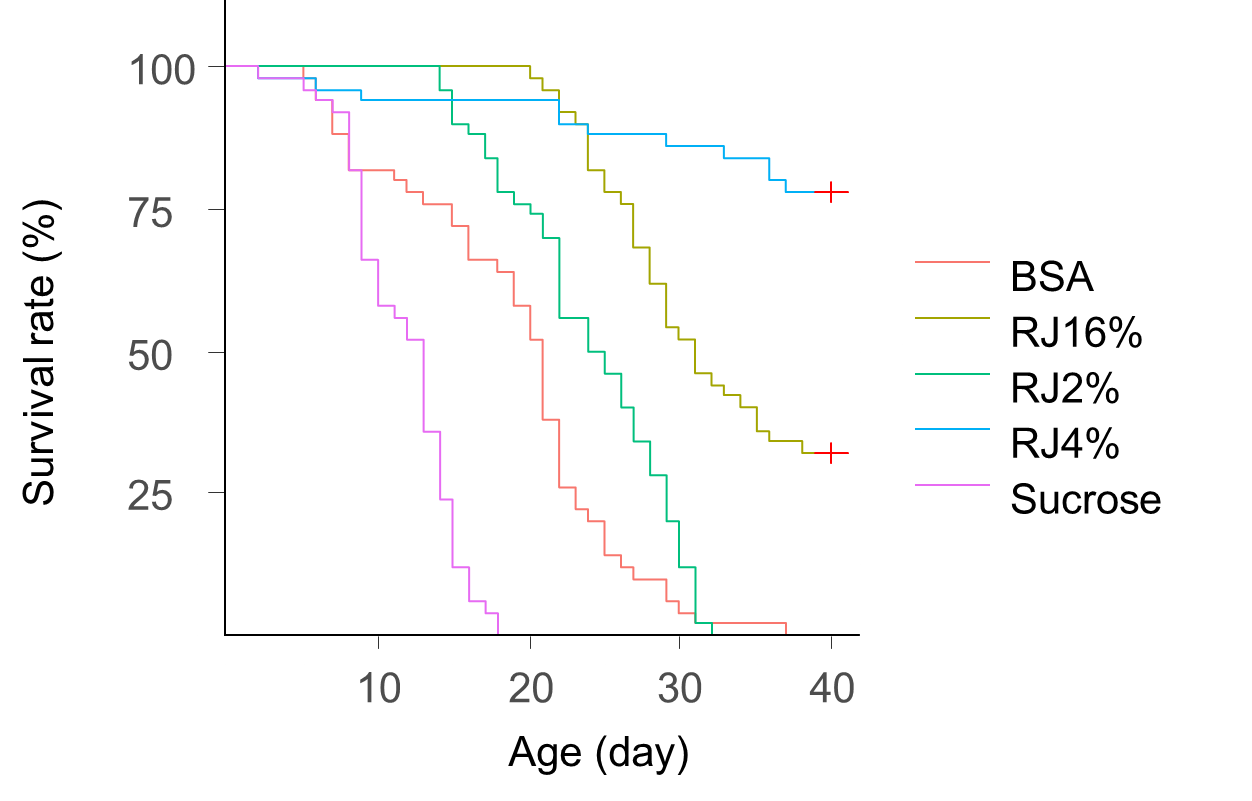


Colony 2: (RJ4% >RJ 16% >RJ 2% > BSA > sucrose)

**Supplemental Fig 1C:**


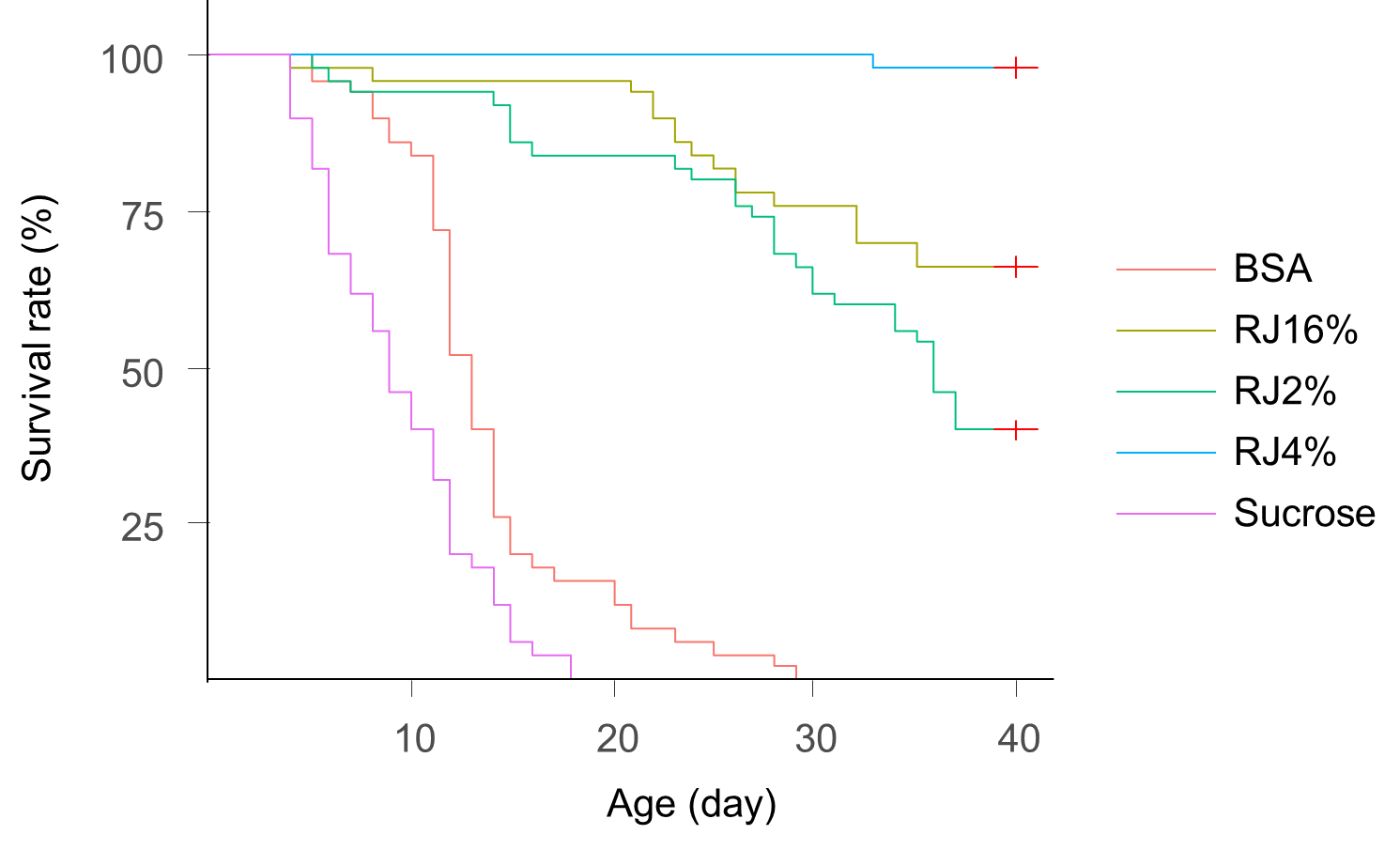


Colony 3: (RJ4% > RJ16% > RJ 2% > BSA > sucrose)

**Supplemental Fig 2A:**

The differences among colonies were not significant except between colony 2 and Colony 3 (X^2^ = 12.2, df = 1, *P* = 0.0005). Colony 1 and 2 were more consistent with the longevity of workers ranked as RJ 4% > *Brassica* = BSA > sucrose, while Colony 2 had them ranked as RJ 4% = *Brassica* > BSA > sucrose (Fig. A and B in S2 Figs). Colony 3 had worker longevity of RJ 4% in the middle and better than sucrose only (Fig C in S2 Figs).


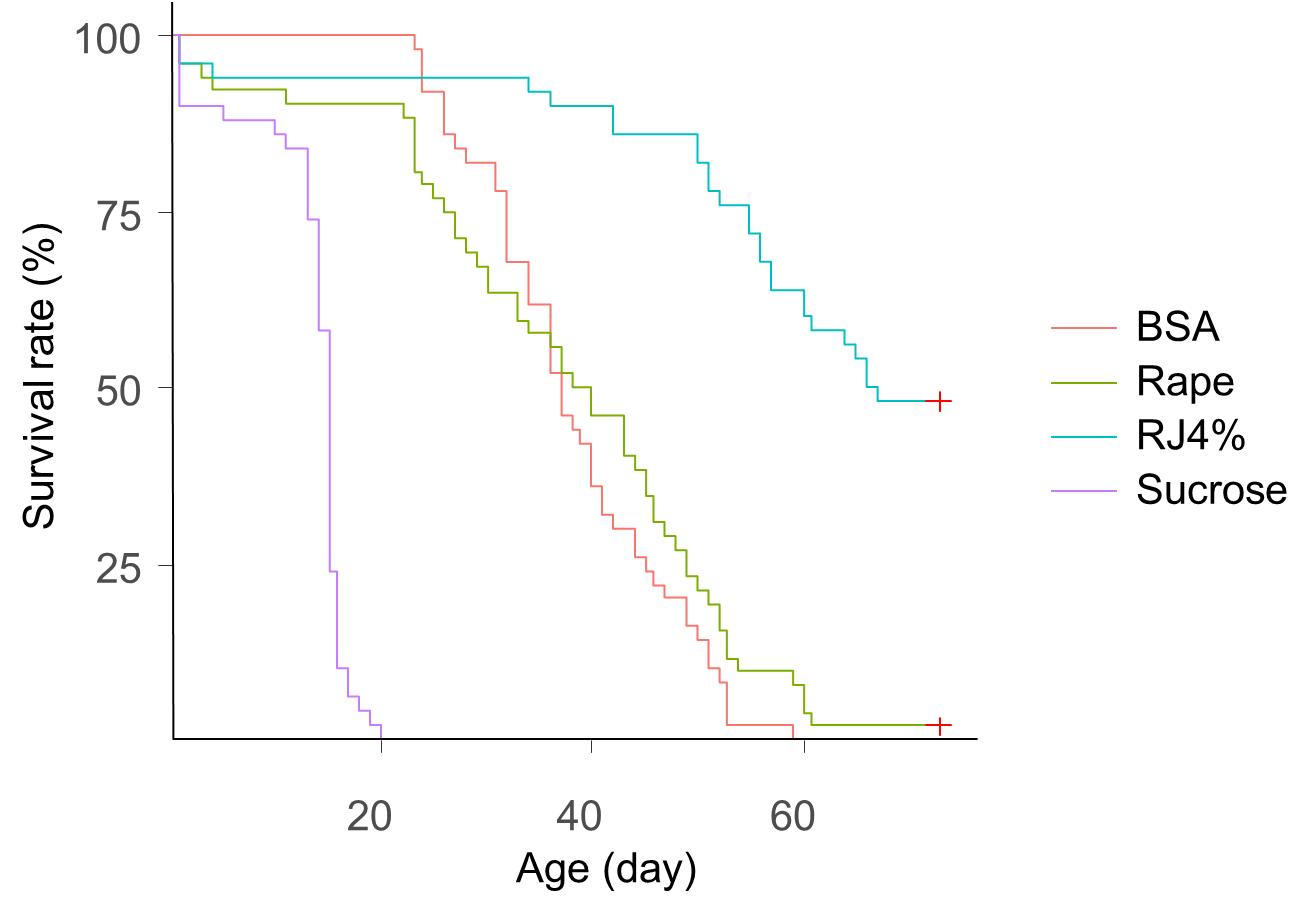


Colony 1: (RJ 4% > rape (*Brassica*) = BSA> sucrose)

**Supplemental Fig 2B:**


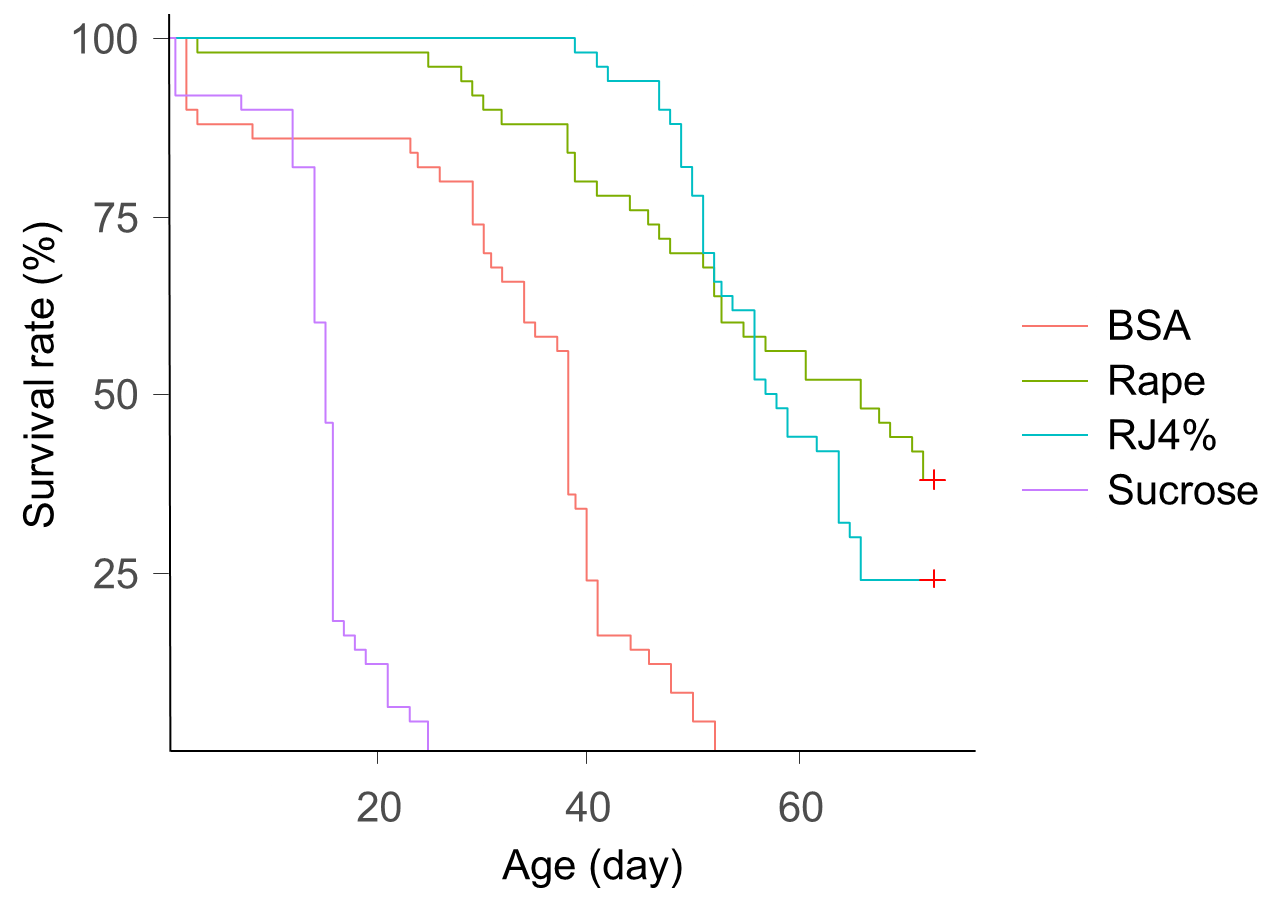


Colony 2: (RJ 4% = rape (*Brassica*) > BSA > sucrose)

**Supplemental Fig 2C:**


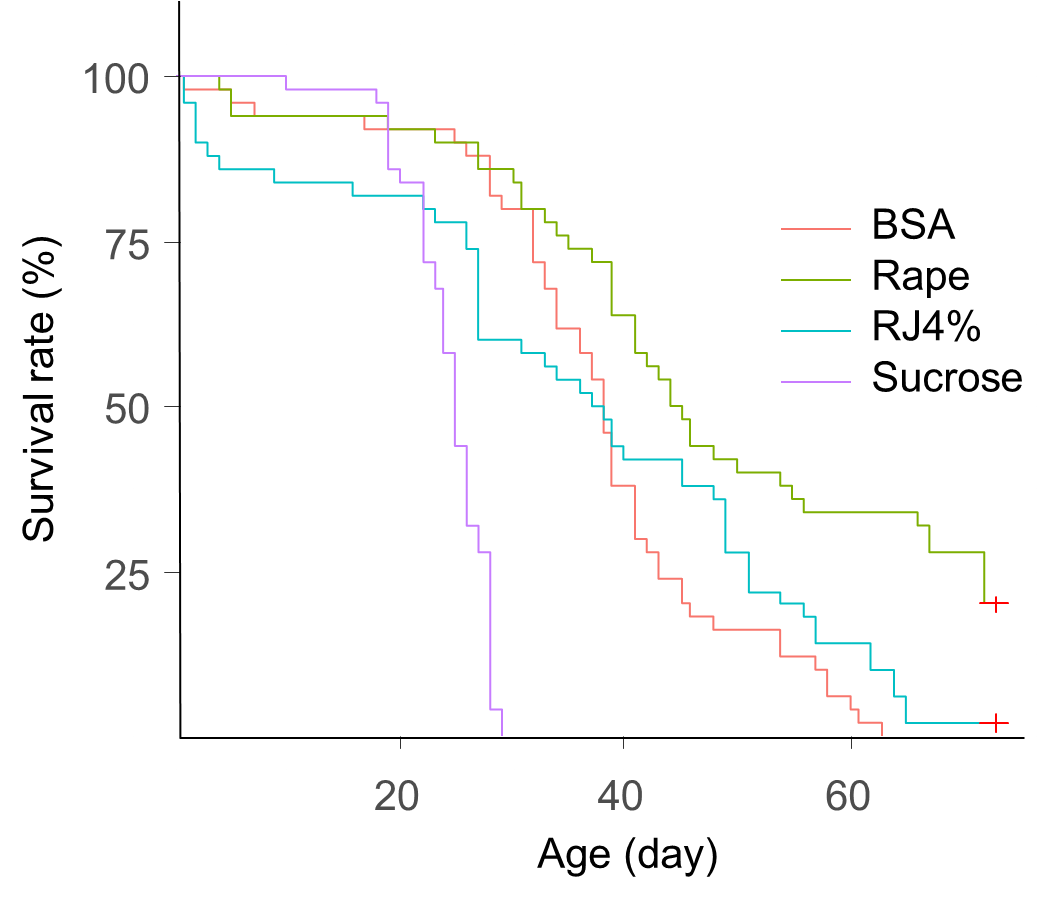


Colony 3: (rape (*Brassica*) > RJ 4% =BSA > sucrose)

**Supplemental Fig 3A:**

The differences among colonies were not significant except between colony 1 and colony 3 (X^2^ = 8.6, df = 1, *P* = 0.003). The differences among the treatments in the three colonies were largely maintained with the rank of worker survival as RJ 4% > RJP_60_ > RJCP > sucrose > RJEE > RJP_30_ with the exception of RJP_60_ equal longevity to RJ 4% and RJCP to Sucrose in colony 3 and having RJEE equal to RJP_30_ in colony1 (Figs in S3 Figs).


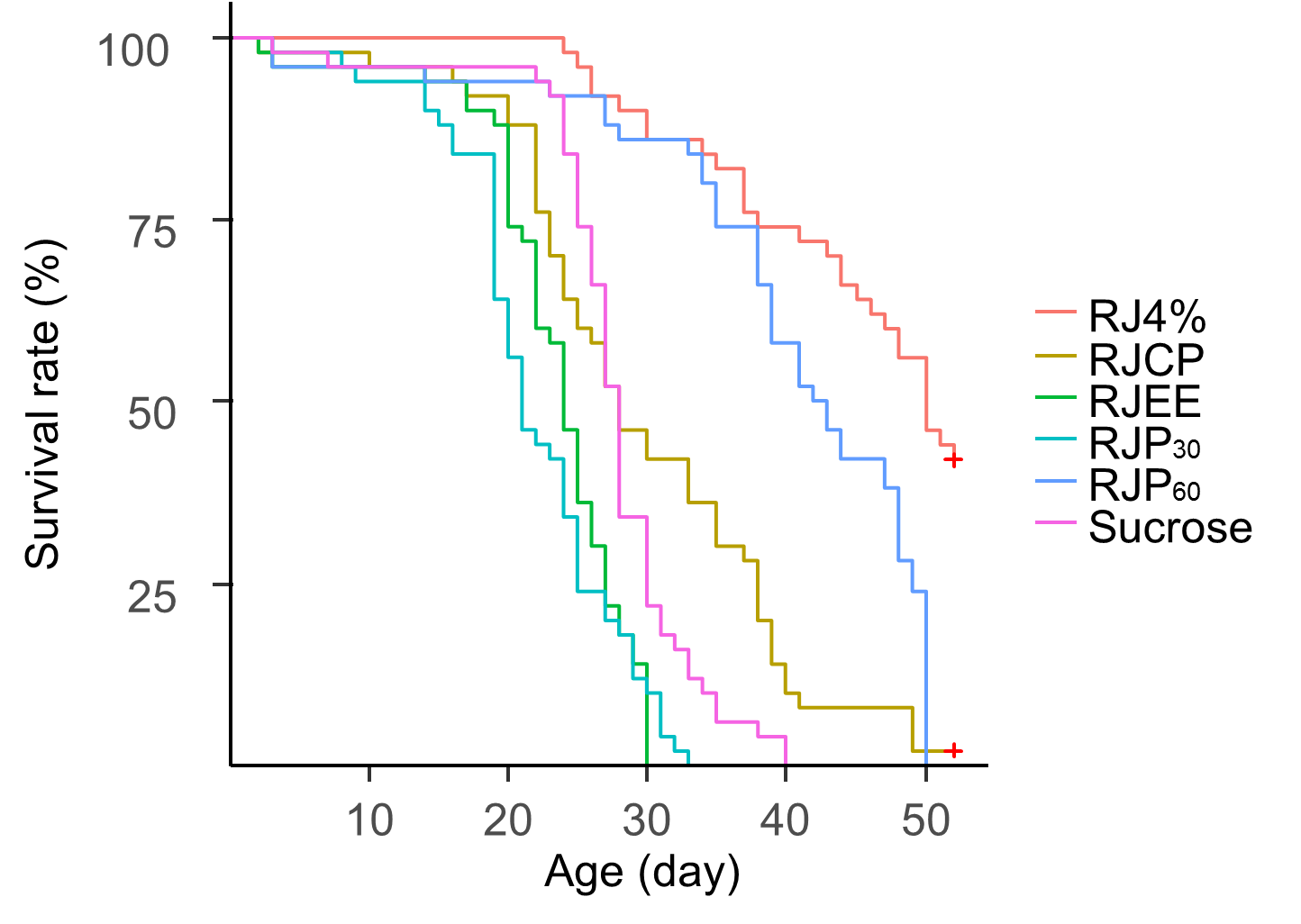


Colony 1: (RJ 4% > RJP _60_ > RJCP > sucrose > RJEE = RJP_30_)

**Supplemental Fig 3B:**


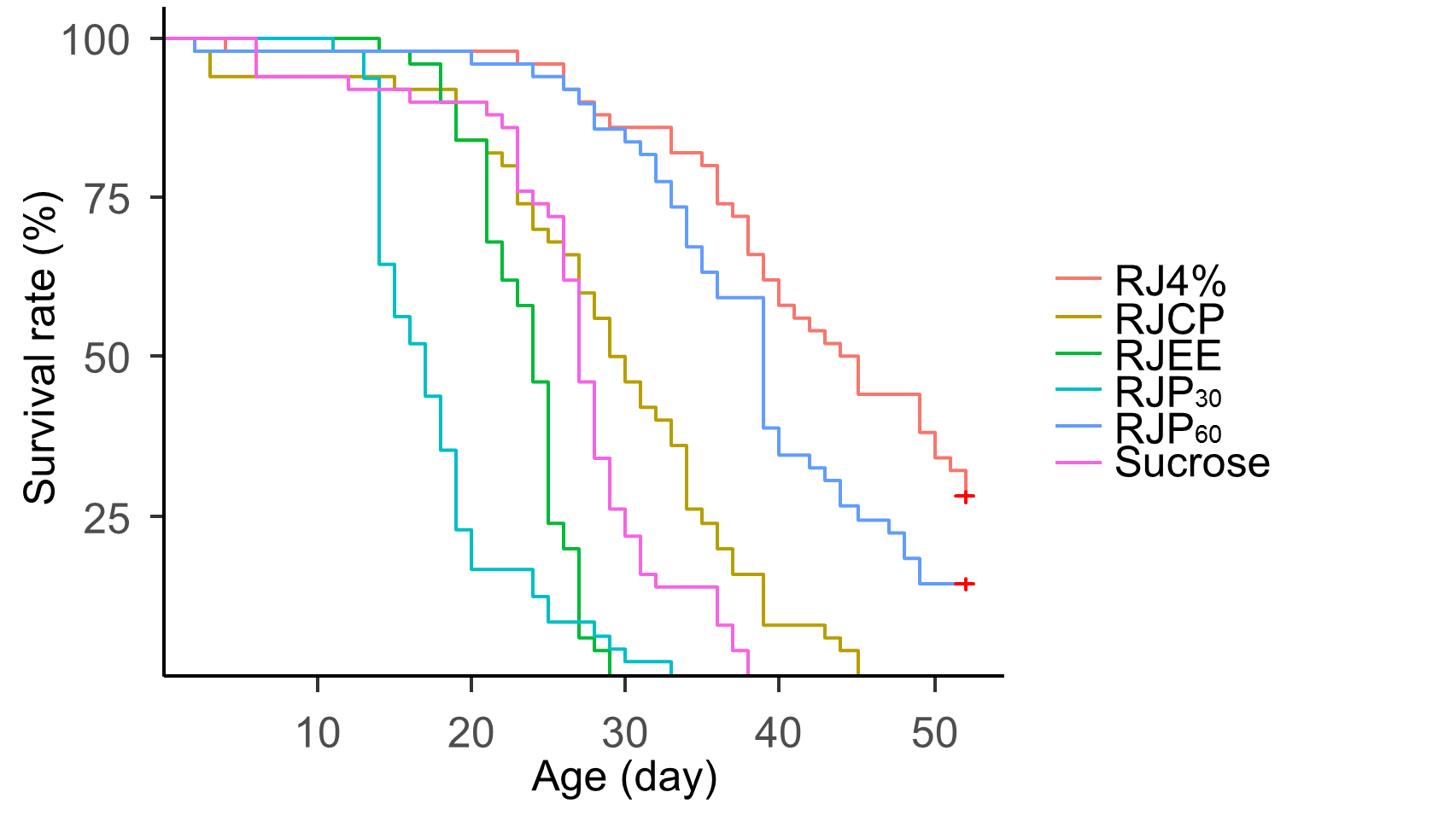


Colony 2: (RJ 4% > RJP _60_ > RJCP > sucrose > RJEE > RJP_30_)

**Supplemental Fig 3C:**


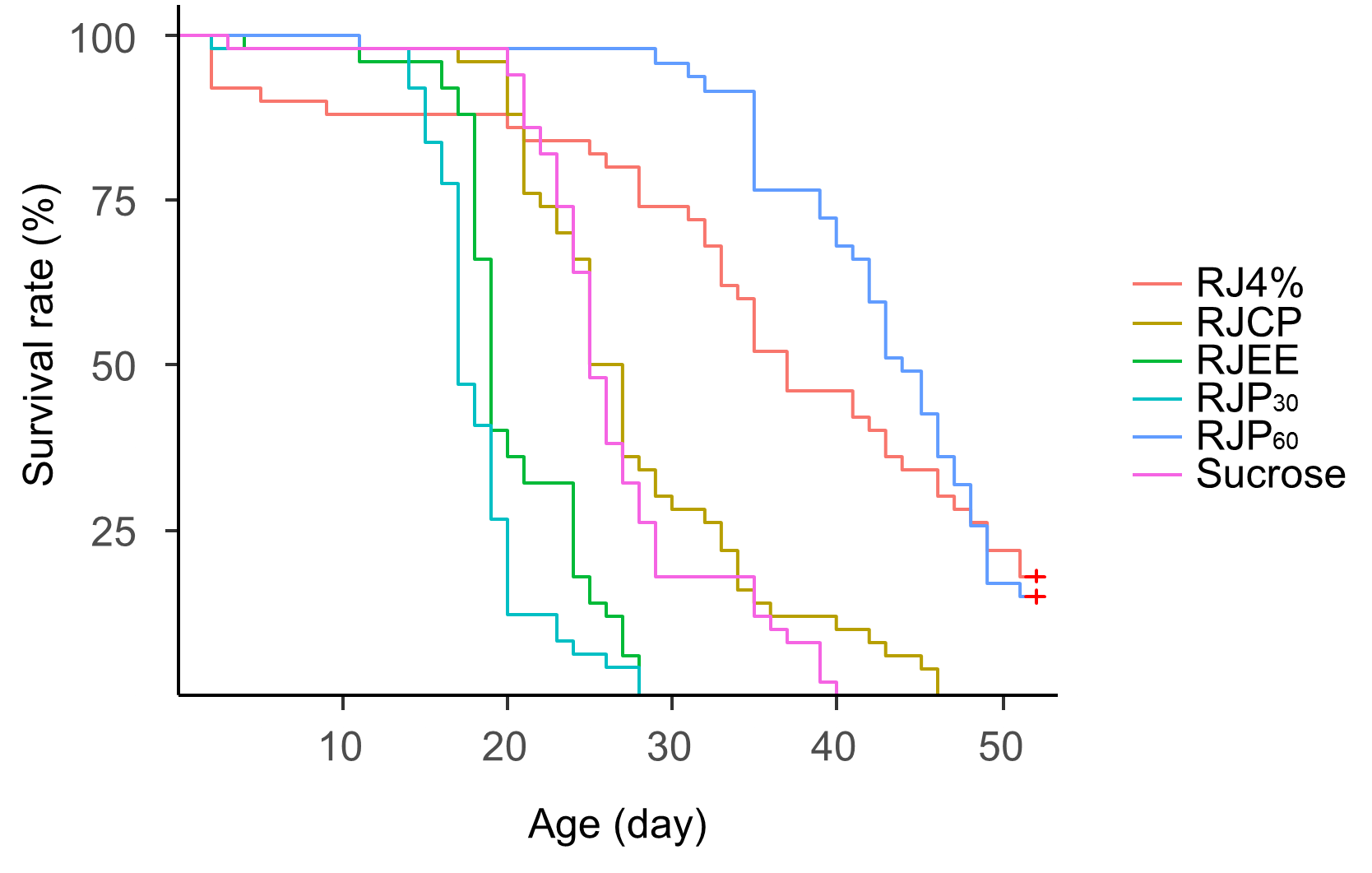


Colony 3: (RJP _60_ = RJ 4% > RJCP > sucrose > RJEE > RJP_30_)
